# A day in the life of mitochondria reveals shifting workloads

**DOI:** 10.1038/s41598-019-48383-y

**Published:** 2019-09-25

**Authors:** Tobias W. Weinrich, Jaimie Hoh Kam, Bill T. Ferrara, Elinor P. Thompson, John Mitrofanis, Glen Jeffery

**Affiliations:** 1 University College Institute of Ophthalmology, London, UK; 2 School of Science, University of Greenwich, London, UK; 3 University of Sydney, School of Medical Sciences, Sydney, Australia

**Keywords:** Biomarkers, Cellular neuroscience

## Abstract

Mitochondria provide energy for cellular function. We examine daily changing patterns of mitochondrial function and metabolism in *Drosophila in vivo* in terms of their complex (I-IV) activity, ATP production, glycolysis, and whole fly respiration in the morning, afternoon and night. Complex activity and respiration showed significant and unexpected variation, peaking in the afternoon. However, ATP levels by contrast are >40% greater in the morning and lowest at night when glycolysis peaks. Complex activity modulation was at the protein level with no evidence for differential transcription over the day. Timing differences between increased ATP production and peaks of complex activity may result from more efficient ATP production early in the day leaving complex activity with spare capacity. Optical stimulation of mitochondria is only possible in the mornings when there is such spare capacity. These results provide first evidence of shifts in cellular energy capacity at the organism level. Understanding their translation may be significant to the chosen timing of energy demanding interventions to improve function and health.

## Introduction

Mitochondria provide the energy that drives much of the physiological function of eukaryotic cells via oxidative phosphorylation and the production of adenosine triphosphate (ATP). They perform a wide range of key metabolic and signaling functions and are critical in regulating the ageing process, even having the ability to trigger cell death^1,2^. They are unique sub-cellular organelles in the animal in having their own DNA (mtDNA) and regularly divide or fuse in response to activity and changing metabolic demand. As with all biological systems, mitochondria have an internal molecular clock with evidence that their dynamics and patterns of gene expression are regulated over the day^3–8^. Disruptions to their molecular clock can lead to alteration in respiration, giving rise to cellular dysfunction and resulting in a decline in health including neuronal degeneration^3,9–12^.

What is not clear from previous studies is whether the activity of mitochondria changes during the course of the day in the whole organism. Does the work-load and productivity of this key organelle change systematically depending on the time of day independent of demand, particularly in relation to complex activity and ATP production that is so critical for cellular function? We explore this question here in *Drosophila* by measuring mitochondrial complex activity, ATP production, whole body respiration and other key metrics at three spaced times over the day. We also tested the spare capacity of mitochondria by stimulating them optically at different time points, linking their available capacity with performance.

## Results

**Figure 1:**
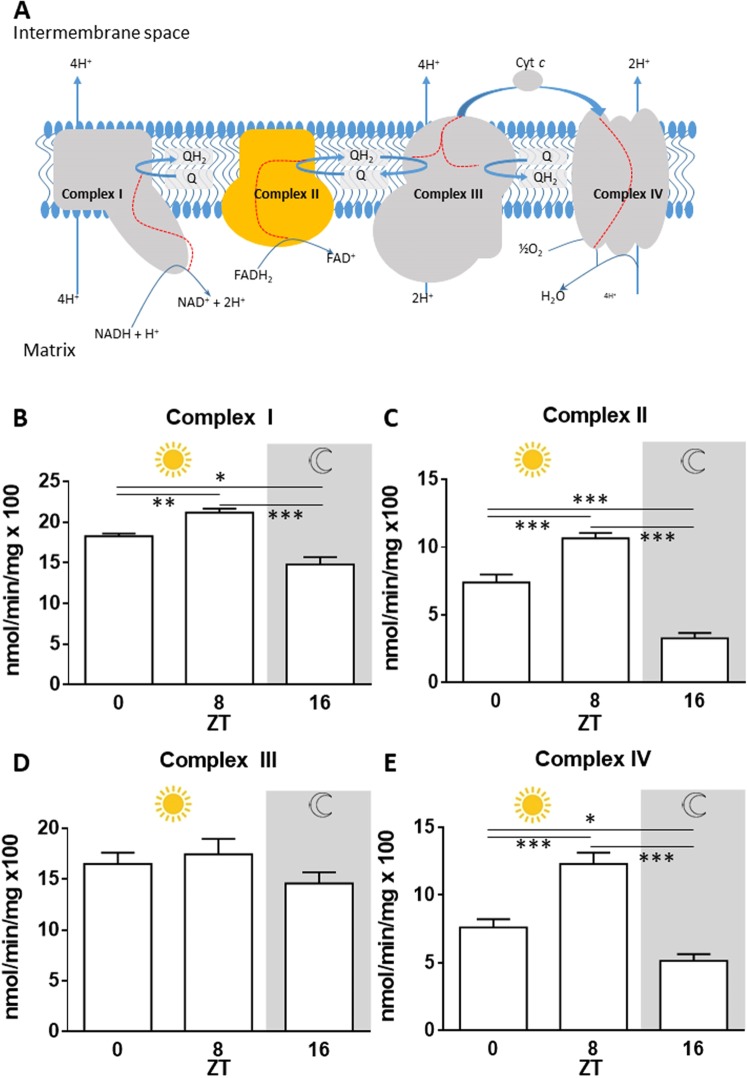
Daily oscillation in mitochondrial electron transport chain (ETC) enzymes. Specific enzymatic activities for electron transport chain enzymes were measured in WT *Drosophila* homogenates collected at indicated times. (**A**) Schematic diagram of the ETC. ETC proteins are embedded in the inner mitochondrial membrane. Electrons enter ETC via complex I & II and transported across complex III to complex IV. The electrons (red lines) provide energy to drive the transport of protons into the inter membrane space, creating an electrochemical gradient. (**B**) Complex I. This peaked at ZT 8 NADH-ubiquinone reductase, ANOVA p < 0.0001, (**C**). Complex II. This peaked at ZT 8, succinate dehydrogenase, ANOVA p < 0.0001, (**D**). Complex III. This peaked at ZT 8, cytochrome c reductase, non-significant ANOVA p = 0.2816, (**E**). Complex IV. This peaked at ZT 8, cytochrome c oxidase, ANOVA p < 0.0001. Gray shading represents the dark phase. Data are presented as means ± SEMs from 6 biological replicates per time-point.

A schematic representation of the complexes and their relationship is shown in Fig. 1A. Data for whole body complex activity in the morning (ZT 0), afternoon (ZT 8) and at night (ZT 16) are shown in Fig. 1B–E. Overall changing patterns in activity are very similar in each complex although they are of differing magnitude. Complexes I, II and IV all show similar significant increases in activity at ZT 8 compared with ZT 0 and ZT 16. In the case of complex II and IV the average increase at ZT 8 compared to ZT 0 and ZT 16 is of the order of 100%. Complex III shows a similar trend although changes here are not statistically significant. Hence, overall complex activity shows significant changes over the day that are consistent between the individual complexes.

**Figure 2:**
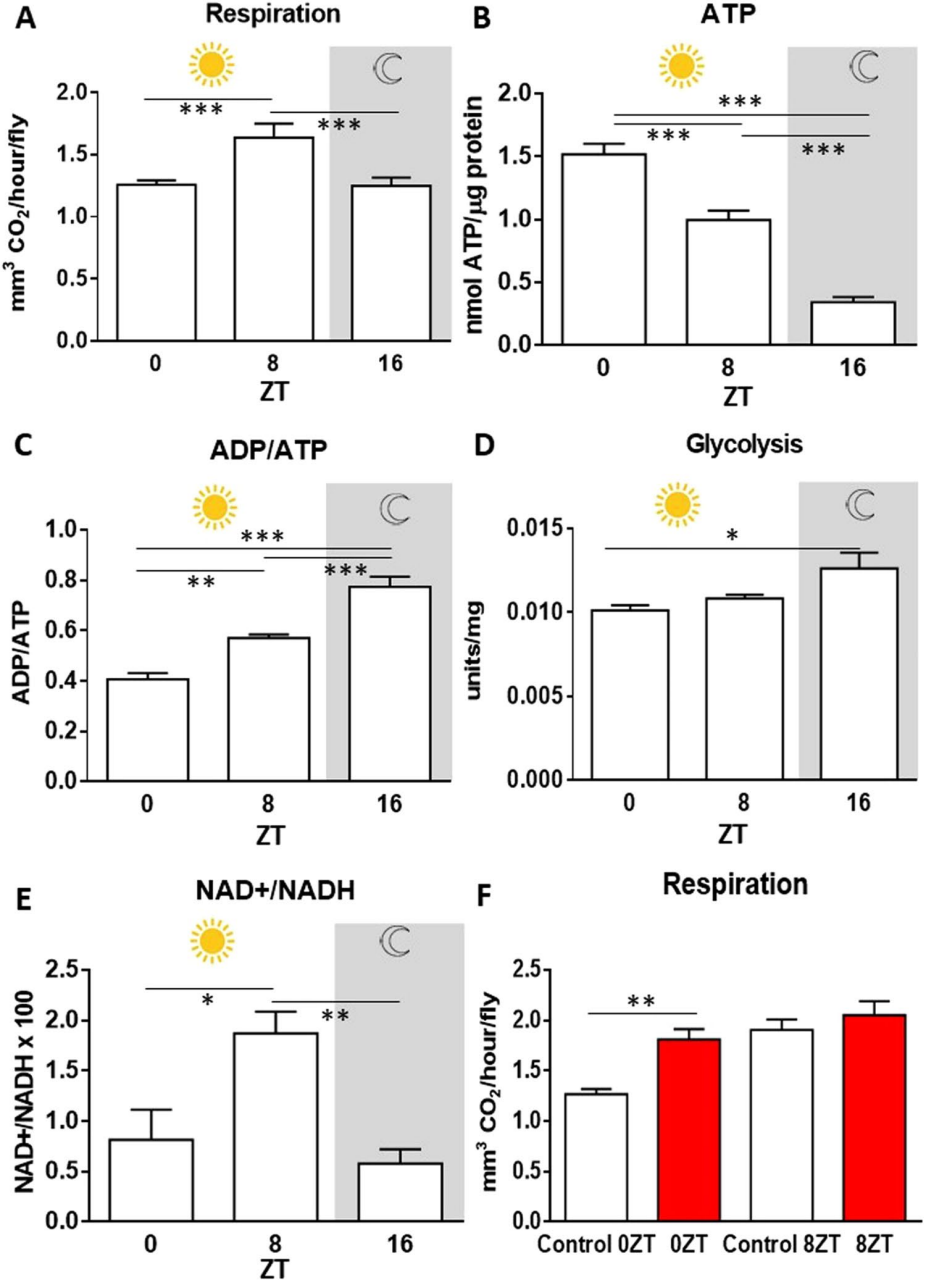
Daily changes in metabolic rate, glycolysis and ATP, ADP/ATP, NAD + /NADH ratio in WT *Drosophila*. Whole body respiration was measured *in vivo*. Whole body ATP, ADP, glycolytic activity were measured in WT *Drosophila* homogenates collected at indicated times. (**A**) Whole body metabolic rate peaked at ZT8, ANOVA p < 0.0017. (**B**) Whole body ATP levels peaked at ZT 0, ANOVA p < 0.0001, (**C**). ADP/ATP ratio, peaked at ZT 16 ANOVA p < 0.0001. (**D**) Glycolytic activity peaked at ZT 16 measured by glyceraldehyde-3-phosphate dehydrogenase enzymatic activity, ANOVA p = 0.0234. (**E**) NAD+/NADH ratio, peaked at ZT 8 ANOVA p < 0.0067. Gray shading represents the dark phase. Data are presented as means ± SEMs from 6 biological replicates per time-point. (**F**) Differences in respiration at different times of the day after 670 nm exposure. 670 nm treated flies are in red and controls are in white. Flies treated for 1 week at ZT8 did not have an increased respiration unlike flies exposed in the morning (ZT0, p = 0.0022, One-way Mann Whitney-U test), where respiration increased significantly. 6 replicates containing 5 flies each.

Figure 2 shows changes in key mitochondrial related metrics at the same time points as those of complex activity revealed in Fig. 1. Here CO_2_ and ATP production, glycolysis and ATP/ADP and NAD/NADH ratios are measured. Whole body cumulative respiration at the three time points was very similar to that found for complex activity, with respiration peaking significantly at ZT 8. The relative increase here was approximately 30% over the other two time periods, ZT 0 and ZT 16 (Fig. 2A). However, measurements of ATP were very different. These were highest at ZT 0 then declined by approximately 40% by ZT 8 and then by >70% at ZT 16 (Fig. 2B). Measurements of ADP/ATP ratios were the opposite of the ATP measurements, with lowest levels found at ZT 0 and then increasing by approximately 40% at ZT 8 and by a similar amount between then and ZT 16 (Fig. 2C). The pattern seen in the ADP/ATP ratio is consistent with the ATP level observation. At ZT0 when ATP levels peak, the ADP/ATP ratio is low, compared to ZT 8, when ATP levels are lower due probably to increased demand. At ZT 16, when the activity of the electron transport chain complexes is lowest, indicative of a low ATP production, the ADP/ATP ratio peaks.

ATP can be generated via mitochondrial respiration or less efficiently, although more rapidly, by glycolysis. Glycolytic activity was also measured at the three time points. It was similar at ZT 0 and ZT 8, the two day time points. However, it increased significantly by approximately 20% at ZT 16 (Fig. 2D). This may possibly be in response to the low levels of complex activity and ATP at this time point.

Significant changes between the time periods were also found in the NAD+/NADH ratios with the changing patterns reflecting those seen in complex activity and respiration. The NAD+/NADH ratio peaks at ZT 8, similar to complex activity and respiration, indicative of a rapid consumption of NADH. At ZT 16 the ratio drops significantly to similar levels found in the morning, with NADH concentration higher than at ZT 8, consistent with increased glycolysis fueling the Krebs cycle (Fig. 2E), but reduced activity in the electron transport chain, which is the main consumer of NADH.

Cytochrome c oxidase in mitochondrial respiration absorbs specific long wavelengths in the deep red to infra-red and this improves mitochondrial function. It increases ATP production and also improves whole body fly respiration as measured in Figure 2A ^13–16^. Hence, if mitochondrial complex activity has spare capacity in the mornings, as suggested by data in Fig. 1 then there should be a difference between the impact of red light (670 nm) on mitochondria between ZT 0 and ZT 8 with the light increasing respiration in the morning (ZT 0) but not in the afternoon (ZT 8). Figure 2F shows that exposure to 670 nm light significantly increases respiration in flies at ZT 0 but not at ZT 8. Here 670 nm treated animals are shown in red histograms and their controls in white. These data support the idea that complex activity lacks spare capacity in the afternoon, while in the mornings spare capacity is present, perhaps because ATP production is relatively high and does not need topping up (Fig. 2B).

**Figure 3:**
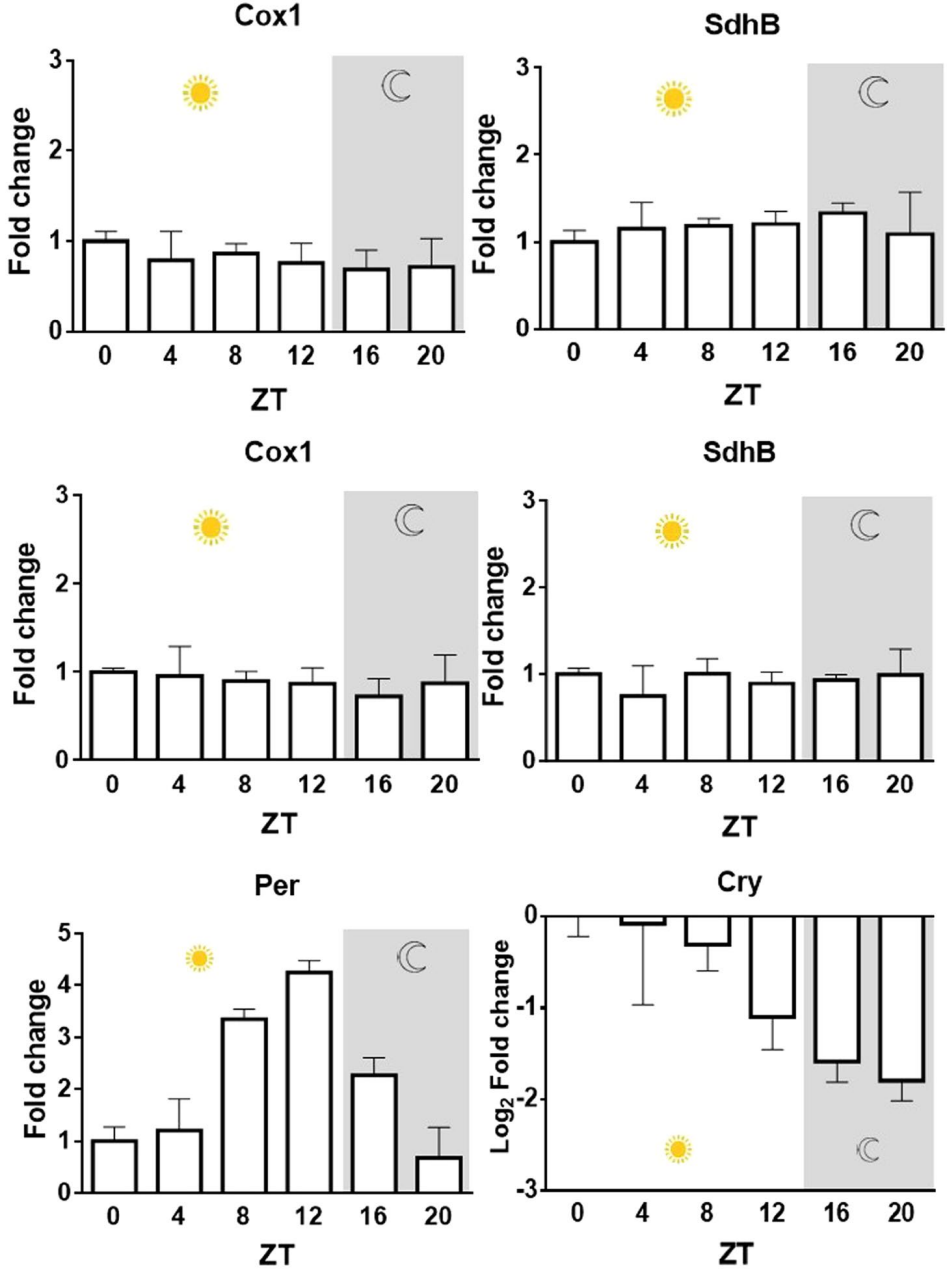
Daily changes in Cox1, SdhB, Per and Cry transcription in WT *Drosophila*. Cox1 and SdhB expression measured in *Drosophila* fly head homogenates at indicated times, and normalised to housekeeping gene glyceraldehyde-3-phosphate dehydrogenase-2 (Gapdh2; **A**,**B**) and mitochondrial large ribosomal RNA (LrRNA; **C**,**D**). Control daily rhythms of Per (**E**) and Cry (**F**) transcription were confirmed in the samples. Fold changes were baselined to ZT 0. One-way ANOVA, Cox1/Gapdh2 p = 0.1640, SdhB/Gapdh2 p = 0.1548, Cox1/LrRNA p = 0.0960, Sdhb/LrRNA p = 0.09476. Data presented are mean ± SEM from three biological replicates (each with three technical replicates) of three experimental repeats.

Changes in complex abundance can either occur as a result of regulation at the transcriptional or protein level. Real time qPCR was undertaken to reveal any regulation of the transcripts of mitochondrion-encoded subunits for which there was evidence for transcriptional rhythmicity in previous studies, or if encoded by the nucleus but with observed rhythmic levels of activity. While not exhaustive, the results revealed no obvious changes that would suggest complexes were regulated at this level whereas *Period (Per)* and *Cryptochrome (Cry)* transcripts showed robust regulation in the samples over 24 h as expected (Fig. 3).

## Discussion

This study shows clear time of day changes in mitochondrial complex activity, ATP levels, whole body respiration and related metrics *in vivo*. Key among these findings is that complex activity and respiration peak at ZT 8 but are relatively low at ZT 0 and ZT 16. The changes in complex activity are modulated at the protein rather than the gene transcription level. However, glycolysis which is not directly a mitochondrial function remains constant during the day (ZT 0 ZT 8) but peaks at night (ZT 16). Here we have chosen to view the entire organism as the unit of interest, rather than a specific organ. This is a relatively novel approach. However, it is possible that our readouts hide other smaller patterns originating from specific organs that are masked within our larger sample.

Previous studies have shown that mitochondria are influenced by daily rhythms^3,4^. However, some of these have employed knock out animals, where aspects of physiological function have been changed, the full consequences of which are difficult to define. Further, there is evidence that the magnitude of circadian events or their control vary significantly between cell lines, organs and species, making global comparisons difficult. This problem is compounded by the adoption of different readout metrics in separate studies. However, one element that does not appear to vary is total mitochondrial content of tissues over the day^3^. The significance of the data we provide is that it is derived from whole animal and without manipulation and spans whole body mitochondrial metrics through to whole body respiratory responses.

The cyclical changes in mitochondrial activity that we reveal are clear and are based on relatively large animal numbers and replicates. In many cases they carry a high level of statistical significance. Consequently they have a high degree of confidence. However, explanations are required for our key findings. Intuitively, one would expect little variation in whole animal complex activity, or that it may be greater at the start of the day when activity is initiated rather than late in the day. An explanation may reside in different mitochondrial workload capacities in relation to available cellar energy. At the start of the day, ATP levels are high and this may result in a homeostatic reduction in complex activity where mitochondria are only required to keep topping up the system, which has not been drained by previous activity. Later, when respiration has increased, complex activity is elevated because ATP has declined, probably due to increased mobility including energy consuming flight.

A test of this hypothesis presents itself with the use of 670 nm light. This light is absorbed by cytochrome c oxidase and increases mitochondrial respiration^13^. This optical manipulation has been used extensively in experimental models of mammalian pathology where mitochondrial function has been undermined and shown to improve outcomes in CNS damage^14^. It has also been used successfully to ameliorate mitochondrial decline in aged *Drosophila* and in bees exposed to insecticides that undermine mitochondrial respiration^15–17^. In our experiments, exposure to 670 nm light improved fly respiration in the mornings but not in the afternoon. This is consistent with mitochondria having reduced spare capacity later in the day when they likely have a heavy workload due to animal activity as reflected in elevated respiration.

We chose *Drosophila* and to explore mitochondrial changes across the whole organism. Although the mouse model, particularly the C57BL/6 has been used, it has confounding issues. They were first inbred by JAX labs in 1921 (https://www.jax.org/news-and-insights/1995/october/microphthalmia-and-ocular-infections-in-inbred-c57-black-mice). Intense in-breeding since has resulted in a faster rate of accumulation of mtDNA replacement substitutions with a different evolutionary pattern than their wild relatives^18^. There is also the fundamental issue of their nocturnal evolutionary history and that this has been subjected to laboratory environments with bright daytime light. While the fly has not suffered from forced shifts from nocturnal to diurnal patterns, the fly model likely suffers from similar long tern inbreeding that may qualify aspects of our results.

There are significant issues that arise from our results. Mitochondrial function and ATP production sustain many aspects of cellular function. Some cellular reactions are highly energy dependent and their efficiency could be influenced by the time of day at which they occur. Understanding how these shifts translate may be significant to the chosen timing of energy demanding interventions to improve function and health and this may be an important avenue of investigation.

## Methods

### Fly husbandry

Male *Drosophila melanogaster* Dahomey were used throughout maintained under standard conditions (12/12 lighting at 25 °C and 70% humidity) and normal food. Newly hatched flies were collected and kept at standard density of 30 male flies in vials (25 × 95 mm) containing 10 mL food medium (changed 3 times per week). Flies were collected at 6 weeks old (±1 day) at the following times: 0, 8 and 16 h post *Zeitgeber* time (ZT), where 0 is light on in the morning and snap-frozen in dry-ice.

### 670 nm treatment

Flies were treated with 670 nm (40 mW cm^2^) for 20 min daily for 7 days, either at 0ZT or 8ZT. Flies were collected after last exposure and assessed for respiration for 2 hours. Light devices were built by C. H. Electronics UK and contained 50 670 nm LEDs (light emitting diode) over 20 cm^2^.

### ATP and ADP

Flies were processed as previously published^19^. Briefly, 5 male flies were homogenised in 100 μl of 6 M guanidine-HCl in extraction buffer (100 mM Tris and 4 mM EDTA, pH 7.8) to inhibit ATPases, followed by freezing in dry ice. The homogenate was then heat treated (95 °C) for 5 min, followed by 3 min centrifugation at maximum speed. The supernatant was collected and diluted (1/50) with extraction buffer. ATP was measured using a commercially available kit based on luminescence (Life Technologies, UK). The relative ATP levels were calculated by dividing the luminescence by total protein concentration (determined by the BCA method). Six replicates containing 5 flies were used for each group. For ADP measurement, ATP was measured, then an ADP-Converting enzyme (Abacam), which converts ADP to ATP was added. The ADP levels are calculated by substracting the second reading from the first.

### Respiration rate

Whole body metabolic rate was assessed by measuring expired CO_2_ production in lab-made respirometers previously described^20^. These contained soda lime, which absorbs expelled CO_2_ produced by the flies, decreasing pressure in the respirometer. Change in gas volume is measured by fluid displacement in a glass capillary attached to the sealed respirometer. In each group there were 6 replicates containing 5 flies each. Because respiration rate is measured over 120 min, the experiment started 1 h before hypothetical collection time, and finished 1 h after.

### Mitochondrial enzymes

We measured enzymatic activity of the complexes in the respiratory chain. Flies were killed at 8 hours intervals, and homogenised. At each time point, 6 replicates were analysed. Flies were homogenised in homogenising buffer (0.121 g of Tris, 0.15 g of KCl and 0.038 g of EGTA in 50 mL distilled water, pH 7.4; 0.854 g of sucrose were added 10 ml the buffer on the experimental day). The homogenate was centrifuged at 300 *g* for 5 min at 4 °C to remove debris. Supernatant was collected, aliquoted and stored at −80 °C for enzyme activity assays. An aliquot was also used to measure protein concentration in each sample following the commercial BCA protein assay (ThermoFisher Scientific) to standardise the amount of protein added in each enzymatic assay. We assayed the activity from 7 biological replicates for each time point post ZT, with the activity of each biological replicate estimated from two technical replicate assays.

### Complex I

Complex I (NADH-ubiquinone reductase) was measured following a dichlorophenolindophenol (DCPIP)-couple method^21^, which is based on the reduction of DCPIP by electrons from decylubiquinol, reduced by complex I after NADH oxidation. This avoids nonspecific NADH oxidation (direct method to assay complex I) interfering in the assay. The DCIP method results in high rotenone sensitivity. Reaction mix contained 25 mM potassium phosphate, 3.5 g/L BSA, 60 μM DCPIP, 70 μM decylubiquinone, 1.0 μM antimycine-A, 0.2 mM NADH and 15 µg/mL sample protein. Reduction of DCPIP is spectrophotometrically monitored at 600 nm and inhibited with rotenone 1 mM.

### Complex II

The catalytic activity of complex II (succinate dehydrogenase) was measured following a standard protocol^22^. Complex II was monitored by following the reduction of DCPIP at 600 nm. The reaction mixture contained 30 mM NaH_2_PO_4_, 100 µM EDTA, 2 mM KCN, 2 µg/mL antimycin A, 2 µg/mL rotenone, 750 µM BSA, 10 mM succinate, 100 µM DCPIP, 100 µM decylubiquinone and 15 µg/mL sample protein, and was inhibited with 400 mM malonate.

### Complex III

Complex III (cytochrome *c* reductase) specific catalytic activity was measured following the increase in reduced cytochrome *c* at 550 nm. The reaction mixture contained 35 mM NaH_2_PO_4_, 2.5 mg/mL BSA, 5 mM MgCl_2_, 2 mM KCN, 2 µg/mL rotenone, 50 µM cytochrome *c*, 25 µM decylubiquinol and 15 µg/mL mitochondrial protein, and was inhibited with 5 µg/mL antimycin A. Potassium borohydride was used to reduce decylubiquione.

### Complex IV

cytochrome *c* oxidase. Specific activity of complex IV was measured following a protocol previously published^22^. Activity was measured by determining the rate of oxidation of reduced cytochrome c at 550 nm. The reaction mixture contained 5 mM MgCl_2_, 2 μg/ml rotenone, 2 μg/ml antimycin A, 1 mM DDM, 45 μM cytochrome c. Each test contained 15 μg of fly homogenate. The reaction was inhibited with 4 mM KCN. Sodium dithionite was used to reduce cytochrome *c*^72^.

### Glycolysis

The reaction kinetics for glyeraldehyde-3-phosphate dehydrogenase (GAPDH) is used as a surrogate marker for glycolysis flux. The specific activity of the GAPDH was measured modifying the procedures of Krebs^23^ and Velick^24^. Enzyme activity was measured by determining the increase in absorption at 340 nm resulting from the reduction of NAD^+^. Briefly, 15 male flies were snap frozen and homogenised in pyrophoshate/arsenate buffer (0.015 M sodium pyrophosphate buffer, pH 8.5 containing 0.03 M sodium arsenate) and total protein content measured with the BCA assay (ThermoFisher Scientific). The reaction mixture contained pyrophosphate/arsenate buffer, supplemented with 0.25 mM NAD^+^, 3 mM DTT/sample, 1.5 ug of protein was added followed by addition of D-glyceraldehyde-3-phosphate (final concentration of 0.25 mM) to start the reaction.

### NAD^+^/NADH Ratio

A hydrazine coupled assay was used to measure NAD^+^ and NADH^25^. Fifteen male flies were killed in dry ice and homogenized in 250 µl of homogenization buffer (10 mM nicotinamide, 10 mM Tris-Cl, 0.05% (w/v) Triton X-100, pH 7.4 adjusted using HCl). The homogenate was centrifuged at 12000 × g for 1 min at 4 C. The supernatant was treated with equal volume of phenol:chloroform:isoamyl alcohol (25∶24:1, v/v), mixed vigorously and centrifuged at 12000 × g for 5 min at 4 °C. The aqueous phase was collected and mixed with an equal volume of chloroform and centrifuged at 12000 × g for 5 min at 4 C. The resulting aqueous phase contains pyridine nucleotide and was used for the assay. Because the enzymatic assay does not distinguish between the oxidised and reduced forms, an additional step is needed. For each sample, two aliquots of 18 µl of the pyridine nucleotide extraction were removed. One of them was mixed with 2 µl of 0.1 M HCl and the other with 2 µl of NaOH so that the final [H + ] or [OH-] were 0.01 M. Both aliquots were heated to 65 °C for 30 min to degrade the reduced or the oxidized pyridine nucleotide respectively. Samples were then immediately placed on ice. Finally, 2 µl of the opposite reagent (NaOH or HCl) was added to neutralize pH. The reaction mixture for the NAD^+^/NADH assay contained: 0.1 M BICINE (N,N-bis(2-hydroxyethyl)glycine), 0.6 M ethanol, 50 mM EDTA, 2 mM PES (phenazine ethosulfate) and 0.5 mM MTT (dimethylthiazol), 0.02% hydrazine and 0.2 mg/ml alcohol dehydrogenase (ADH). For the assay, a NAD^+^ standard curve was used (100-50-25-12.5-6.25-3.125-1.57-0 µM). 5 µl of standard or sample were added to each well containing 120 µl of reaction mixture without PES and MTT, which were added to start the reaction together with ADH before the plate was read at 570 nm. Kinetic curves were taken for the first 5 min to measure reaction velocity. The NAD^+^ standard was used to determine the concentration of NAD^+^ and NADH in each sample.

### RNA extraction and qPCR

RNA was extracted from 80–100 *Drosophila* heads/sample, with 50 heads weighing approximately 50 mg. These were homogenised in 1 mL of TRIzol Reagent (Ambion, UK) using micro pestles (Sigma). Homogenate was incubated at RT for 5 min. 0.2 mL chloroform was added, shaken vigorously (15 s) and incubated at RT for 3 min. Samples were spun at 12.000 *g* 15 min at 4 °C. Aqueous phase was collected and RNA was purified using RNeasy mini kit (Qiagen, UK). Genomic DNA was removed using On-Column DNase (Sigma). Purity of eluted RNA was estimated by the OD ratios (A_260_/A_280_ > 2.0) and quantified using the BioDrop spectrophotometer (BioDrop, UK), then stored at −80 °C until needed. 70 ng total RNA from each sample was converted into cDNA using RevertAid First Strand cDNA Synthesis Kit (ThermoScientific) with random hexamer primers as reverse transcription primers. Three biological samples were analysed in triplicate using gene specific primers, at designated ZT times. Transcription levels were determined using the Step One PCR machine (Applied Biosystems, CA, USA) with the SYBR Green probe (Qiagen). Prepared cDNA was amplified under the following conditions: 1 µL of cDNA, 5 µL of 2X QuantiFast SYBR Green PCR master mix (ThermoFisher Scientific), 1 µL of each forward/reverse primer (10 pmol) and 3 µl of H_2_O per 10 µL reaction. The PCR were as follows: initial denaturation 95 °C 5 min, 40 cycles of 95 °C, 30 sec and 58 °C, 1 min. Primers were designed using OligoArchitect (Premier Biosoft International, California, USA) to produce amplicons between 100–200 bp. PCR and gel electrophoresis with the prepared cDNA was used to verify primer specificity (i.e., single product) and to rule out any genomic DNA contamination (Table 1). Melt curves were analysed to ensure single melt curve peaks were produced. Primer efficiency (E) and primer stability (M) were determined over 10-fold serial dilutions of cDNA. Primer efficiencies were determined such as E = (10(−1/slope)−1) * 100, high amplification efficiency being between 90–120%^26^. Primer expression stability was calculated using NormFinder software^27^ and geNorm software^28^. qPCR transcription levels were calculated according to the ΔΔCt-methods^29^. Data were analysed in Graphpad Prism 7.0 (La Jolla, California, USA) and subjected to either student t-test or one-way ANOVA as stated in legends. With significant results in a one-way ANOVA, post-hoc Dunnett tests were performed to determine degree of significance^30^.

**Table 1:** Primers for qRT-PCR. Oligonucleotide primer pairs used for RT-PCR amplification and quantification of transcripts known to lack nuclear pseudogenes for respiratory complex subunit, housekeeping gene and circadian control gene transcripts (mitochondrion-encoded gapdh2, glyceraldehyde-3-phosphaste dehydrogenase FBgn0001092; lrRNA, mitochondrial large rRNA FBgn0013686, cox1, coil cytochrome c oxidase subunit II FBgn0013675, nucleus-encoded sdhB, succinate dehydrogenase, subunit B FBgn0014028 and per, period and cry, cryptochrome.

Target	Forward sequence (5′ to 3′)	Reverse sequence (5′ to 3′)
gapdh2	CGTTTCTACCGATTTCCT	GTTGTCGTACCAAGAGAT
lrRNA	GTCTAACCTGCCCACTGA	TTCGTCCAACCATTCATTCC
cox1	TATTAGATGTTGATAACCGAGTAG	TAATCGTCCAGGTGTACC
sdhB	CTGTACGAGTGCATCCTG	GGTCCTTCAACTTGTTCAGA
per	GGGTCCTGGAAACGAGTGAG	GAATGGTGACATCCCACGG
cry	TTACTTCTGTTGGATGAG	AACTGGACATTCTTGAAG

### Statistics

Data was acquired and analysed with GraphPad Prism v6.0 ANOVA was used in cases of multiple comparisons. Differences were considered significant at p < 0.05 as noted in the figure legends^30^.

## Data Availability

Any additional data required will be made available on request.
